# Role of Semaphorin 3A in Kidney Development and Diseases

**DOI:** 10.3390/diagnostics13193038

**Published:** 2023-09-25

**Authors:** Yizhen Sang, Kenji Tsuji, Hiroyuki Nakanoh, Kazuhiko Fukushima, Shinji Kitamura, Jun Wada

**Affiliations:** 1 Department of Nephrology, Rheumatology, Endocrinology and Metabolism, Okayama University Graduate School of Medicine, Dentistry and Pharmaceutical Sciences, 2-5-1 Shikata-cho, Okayama 700-8558, Japan; yizhensang@outlook.com (Y.S.);; 2 Department of Rheumatology and Immunology, Shanghai Tenth People’s Hospital, Tongji University School of Medicine, Shanghai 200072, China; 3 Center for Systems Biology, Program in Membrane Biology, Division of Nephrology, Department of Medicine, Massachusetts General Hospital and Harvard Medical School, Boston, MA 02114, USA; 4 Department of Nursing Science, Faculty of Health and Welfare Science, Okayama Prefectural University, Okayama 719-1197, Japan

**Keywords:** semaphorin 3A, neuropilin-1, podocyte, diabetic nephropathy, acute kidney injury, chronic kidney injury, lupus nephritis, fibrosis, apoptosis, inflammation

## Abstract

Kidney diseases are worldwide public health problems affecting millions of people. However, there are still limited therapeutic options against kidney diseases. Semaphorin 3A (SEMA3A) is a secreted and membrane-associated protein, which regulates diverse functions, including immune regulation, cell survival, migration and angiogenesis, thus involving in the several pathogeneses of diseases, including eyes and neurons, as well as kidneys. SEMA3A is expressed in podocytes and tubular cells in the normal adult kidney, and recent evidence has revealed that excess SEMA3A expression and the subsequent signaling pathway aggravate kidney injury in a variety of kidney diseases, including nephrotic syndrome, diabetic nephropathy, acute kidney injury, and chronic kidney disease. In addition, several reports have demonstrated that the inhibition of SEMA3A ameliorated kidney injury via a reduction in cell apoptosis, fibrosis and inflammation; thus, SEMA3A may be a potential therapeutic target for kidney diseases. In this review article, we summarized the current knowledge regarding the role of SEMA3A in kidney pathophysiology and their potential use in kidney diseases.

## 1. Introduction

Semaphorins are guidance proteins regulating cellular morphology and functions, and, thus, have important roles in developments and diseases, including cancers and metabolic diseases [1]. Semaphorin belongs to the super semaphorin family, consisting of 30 glycoproteins and divided into eight classes [2]. Class 1 and class 2 semaphorins have been identified in invertebrates, and class 3–7 semaphorins in vertebrates. In vertebrates, class 3 semaphorin (SEMA3A-3G) is the only member of secreted proteins [3]. In 1990, Raper et al. isolated a molecule from embryonic chick brain that induced the collapse of neuronal growth cones in culture, and the molecule was originally named Collapsin [4,5], but it was renamed as SEMA3A. Since then, SEMA3A has been well studied not only in axon guidance, but also in pleiotropic functions, including in angiogenesis, immune cell regulation and cell migration [6]. SEMA3A is expressed in kidneys, as well as the nervous system, heart, lung, eyes, bone and immune cells [7,8,9,10,11,12], and regulates tissue development and the maintenance of homeostasis, for example, through the regulation of cell proliferation and migration, and the immune system [13,14,15]. Several reports have identified an increase or decrease in SEMA3A expression under several disease conditions [16,17,18,19]; thus, SEMA3A has been suggested as a possible biomarker, as well as a therapeutic target. For example, it has been reported that retinal SEMA3A expression was increased in a retinal-vein-occlusion mouse model, where the anti-SEMA3A neutralizing antibody BI-X mediated a protective role in intraretinal edema and retinal blood flow [12]. A SEMA3A inhibitor was also reported to protect retinal ganglion cells in animal models of optic nerve injury, retinal ischemia and glaucoma [13]. In a neuron system, the inhibition of SEMA3A enhanced functional recovery during the subacute-stroke recovery period [20]. SEMA3A not only regulated the organization of brain structures affected by autism spectrum disorder (ASD), but also was related to neuron inflammatory processes in ASD [21]. Moreover, lipopolysaccharide (LPS)-induced vascular endothelial cell activation, vascular inflammation, and vascular oxidative stress were substantially improved by the inhibition of SEMA3A using siRNA [22]. The serum SEMA3A level was proposed as a biomarker for diabetic retinopathy in patients with type 2 diabetes [23]. Importantly, it also reflected the severity of diabetic retinopathy, suggesting a value for evaluating patients’ prognosis. A quantitative real-time PCR analysis using glioma tissues verified that SEMA3(A–G) together with six other genes may be useful as biomarkers in the prognosis of a glioma patient’s outcome [24]. Serum SEMA3A was decreased in patients with systemic lupus erythematosus (SLE) and was increased in patients with rheumatoid arthritis and Sjogren’s syndrome, suggesting the important role of SEMA3A in immune-related diseases [25]. SEMA3A expression in cancer tissues was shown to be an independent prognostic factor of overall survival for various types of cancer, such as oral cancer, gastric cancer, breast cancer, prostate cancer, glioblastoma and ovarian cancer. Of note, SEMA3A was significantly correlated with the stage and grade of the disease, depth of invasion, presence of metastases and survival [26,27]. In addition, recent accumulated evidence has also revealed important roles of SEMA3A in kidney diseases.

Kidney diseases, characterized as kidney dysfunction, are a growing public health burden, with a huge economic cost on healthcare systems worldwide [28]. There are several important causes for kidney diseases, including diabetes mellitus (DM), hypertension, glomerulonephritis, obesity and aging. In 2017, a survey showed that 697.5 million cases of all-stage chronic kidney disease (CKD) were recorded, for a global prevalence of 9.1% [29]. Therefore, elucidating the complicated molecular pathology of kidney disease is an urgent task for developing new therapeutic strategies to manage patients with kidney diseases. Over the past few decades, many efforts have been devoted to investigating and evaluating the progression of CKD. One of the important advents for CKD therapy has been the use of sodium–glucose co-transporter-2 (SGLT-2) inhibitor [30,31], which slows down CKD progression. In addition, glucagon-like peptide (GLP) 1 receptor agonists have been reported to reduce the incidences of kidney events in patients with DM [32]. Several promising therapies for CKD, including apoptosis signal-regulating kinase 1 (ASK1) inhibitor, endothelin receptor antagonist, phosphodiesterase inhibitor, Janus kinases (JAK)1/2 inhibitor and nuclear factor erythroid 2-related factor 2 (Nrf2) activator have been undergoing clinical trials [33,34,35,36,37]. Nevertheless, its etiology and pathogenesis are still far from being elucidated. Accumulated evidence has suggested importance roles of SEMA3A signaling in kidney development and several kidney diseases, including proteinuric diseases, acute kidney injury (AKI) and CKD. Several reports have indicated the potential use of inhibitory drugs of SEMA3A signaling against kidney diseases, and also identified SEMA3A expression as a potential biomarker for the early detection and prognosis of AKI and the relapse of nephrotic syndromes. In this review article, we summarize the current body of evidence about SEMA3A in kidney development and diseases.

## 2. SEMA3A and Its Receptors, and Its Expression in Kidneys

**Figure 1:**
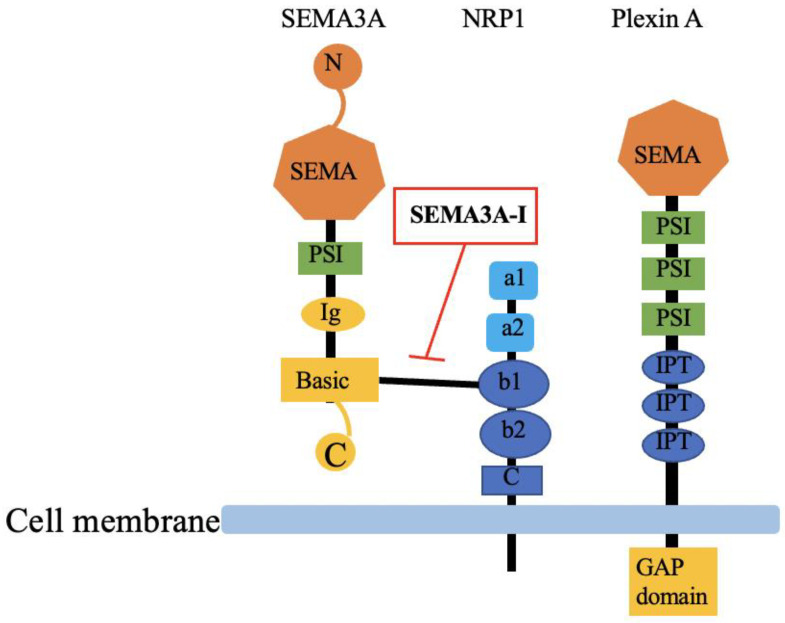
Structure of SEMA3A and its receptors, NRP1 and Plexin A. SEMA3A: semaphorin3A; SEMA3A-I: semaphorin3A inhibitor; NRP1: neuropilin-1; N: N-terminal; SEMA: SEMA domain; PSI: cysteine-rich plexin–semaphorin–integrin domain; Ig: immunoglobulin-like domain; Basic: basic domain; C: C-terminal; a1: complement binding domain a1; a2: complement binding domain a2; b1: coagulation factor V homology domain; b2: coagulation factor VIII homology domain; C: c-domain; IPT: Ig-like, plexin and transcription factor domain; GAP domain: GAP domain GTPase activating protein.

SEMA3A, as with other semaphorin family members, has a conserved N-terminal SEMA domain that contains 500 amino acids, and a diverged C-terminus. The central feature of the SEMA domain is a disulfide-rich seven-blade β-propeller fold. A small cysteine-rich plexin–semaphorin–integrin (PSI) domain is between the SEMA domain and the immunoglobulin (Ig)-like domain. The basic domain is located at the C-terminus of SEMA3A. The C-terminus determines its affinity to neuropilin-1 (NRP1) (Figure 1) [3,38]. SEMA3A has two receptors: NRP1 is necessary for ligand binding, while plexin A is important to subsequent signal transduction. NRP1 is a transmembrane protein with a molecular weight of 120–130 kDa. The extracellular part contains three kinds of domains, made up of two complement binding domains a1/a2, two coagulation factor V/VIII domains, and a c-domain, and is connected to the NRP1 cytoplasmic part (Figure 1) [3]. The signaling receptor Plexin A is a transmembrane glycoprotein with a SEMA3A-like extracellular part, which, implying their common evolutionary origin, is followed by three PSI domains and three IPT (Ig-like, plexins and transcription factors) domains. Importantly, plexin is the only receptor possessing a highly conserved GAP domain (GTPase activating protein), regulating diverse functions in cell activity. Plexin A downstream signaling is complex, involving the following GTPases: R-Ras, M-Ras and Rap (from the Ras family), RhoA, Ras-related C3 botulinum toxin substrate 1 (Rac1), and Rnd (from the Rho family) as well as Fyn phosphorylation [39]. During signal transduction, the SEMA3A–NRP1–Plexin complex forms a holoreceptor [40]. The three-dimensional structure of the SEMA3A–NRP1–Plexin complex has recently been reported [41]. Together, their characteristics add more crosstalk to the pathways involved in SEMA3A signaling. It has been reported that pleiotropic signaling pathways are involved in SEMA3A-–NRP1–Plexin A signaling, including c-Jun N-terminal kinase (JNK) and the Akt signaling pathway.

It has been reported that excess SEMA3A expression may accelerate kidney injury in a variety of kidney diseases via the JNK or Akt signaling pathway [42,43,44,45,46], suggesting that targeting SEMA3A signaling may be a potential therapy against kidney injury. In mammalian adult kidneys, SEMA3A is expressed in podocytes and tubular cells [47], while SEMA3A receptors, NRP1 and Plexin A are expressed throughout life in podocytes, endothelial cells and tubular cells [48,49]. In addition, a recent analysis using a single-cell RNA sequence revealed increased NRP1 expression in activated fibroblasts, suggesting a possible involvement of SEMA3A signaling during the progression of kidney fibrosis [50]. NRP1 is also known as a co-receptor for the vascular endothelial growth factor A isoform 165 (VEGF_165_); thus, SEMA3A plays a role in suppressing angiogenesis by the competitive inhibition of VEGF signaling through the inhibition of binding to NRP1 [51]. It has been reported that a SEMA3A inhibitor (SM-345431) inhibited connections between SEMA3A and NRP1, thereby blocking SEMA3A signaling [46,52] (Figure 1). Indeed, SM-345431 was shown to antagonize SEMA3A-induced axonal-growth-cone collapse in embryonic neurons [53]. In addition, SM-345431 was shown to preserve the corneal nerve and epithelial integrity in a rodent dry-eye model [54]. To specify an interaction between a peptide inhibitor and the SEMA3A–NRP1 system, a peptide inhibitor was modified with the photoactivatable amino acids-4-benzoyl-l-lphenylalaine or photo-l-leucine using solid-phase peptide synthesis. SEMA3A–peptide interaction was found in a defined area of the SEMA domain, which was also involved in NRP1 [55].

## 3. SEMA3A in Kidney Development

SEMA3A has roles in axon pathfinding, and cardiovascular, lung and kidney patterning during organogenesis [56,57]. During kidney development, SEMA3A plays an important role in patterning the ureteric bud branching. Recombinant SEMA3A decreases the number of developing glomeruli in vitro and inhibits ureteric bud branching via the downregulation of glial-cell-line-derived neurotrophic factor (GDNF) signaling, competition with VEGF_165_ and decreased activity of Akt survival pathways. Conversely, the deletion of SEMA3A in mice enhances ureteric bud branching [58]. Thus, SEMA3A functions as a negative regulator of ureteric bud branching during normal kidney development. Reidy et al. established loss- and gain-of-function mouse models, which revealed that SEMA3A^−/−^ mice showed increased endothelial cells and defects in renal vascular patterning, whereas SEMA3A^+/+^ mice had normal wide-open capillary loops, observed using light microscopy. The morphometric analysis of transmission electron microscopy (TEM) revealed that SEMA3A^−/−^ mice had effaced podocyte foot processes, which were associated with albuminuria. On the other hand, the overexpression of podocyte SEMA3A resulted in glomerular hypoplasia, undifferentiated podocytes and congenital proteinuria [48]. Taken together, a balanced SEMA3A expression may be essential for normal glomerular development, glomerular-filtration-barrier function and ureteric bud branching. In addition to kidney development, the role of SEMA3A in matured kidneys has also been explored. Recombinant SEMA3A injection in adult mice induced nephrotic-range proteinuria [44]. In addition, it was also reported that excess SEMA3A increased starvation-induced apoptosis in cultured podocytes and in the developing kidney in vivo [48,49]. Taken together, SEMA3A not only regulates kidney development, but also may be involved in kidney diseases.

**Table 1:** Expression of SEMA3A in kidney diseases.

Disease	Etiology	Species	Sample	SEMA3A Expression	Ref.
Proteinuric diseases	MCNS	Human	Urine	Increase	[59]
	PAN	Rats	Kidney	Increase	[57]
DN	-	Human	Urine	Increase	[60]
	db/db	Mice	Kidney	Increase	[60]
	db/db	Mice	Kidney	Increase	[57]
	Streptozotocin	Mice	Kidney	Increase	[61]
AKI	IRI	Mice	Kidney	Increase	[62]
	LPS	Mice	Kidney	Increase	[45]
	Contrast	Human	Urine	Increase	[63]
	Cardiac operation	Human	Serum/Urine	Increase	[43]
CKD	-	Human	Urine	Increase	[64]
LN	-	Human	Kidney	Increase	[65]
	-	Human	Urine	Decrease	[66]

DN: diabetic nephropathy; AKI: acute kidney injury; CKD: chronic kidney disease; LN: lupus nephritis; MCNS: minimal-change nephrotic syndrome; PAN: puromycin; IRI: ischemia-reperfusion injury; LPS: lipopolysaccharide.

## 4. SEMA3A and Kidney Diseases

### 4.1. Podocytopathy and Diabetic Nephropathy

Podocytes, which have crucial roles in the kidney filtration barrier, line out of the glomerular basement membrane (GBM), prevent urinary protein loss [67]. Podocyte foot processes are linked by slit diaphragms, which regulate cell shape and work as a filtration barrier [68]. Hence, podocyte injury is associated with proteinuria. As indicated above, recombinant SEMA3A injection into adult mice induced nephrotic-range proteinuria [44]; the increase was within 4 h and was resolved within 24 h. TEM analysis revealed the extensive fusion and effacement of podocyte foot processes in kidneys examined 4 h after SEMA3A injection, which were recovered at 48 h, demonstrating that excess circulating SEMA3A may cause podocyte ultrastructural abnormalities, and the permeability of the glomerular filtration barrier is transient and reversible, providing proof of the principle of excess SEMA3A and glomerular disease. In addition, it has also been reported that the SEMA3A-induced downregulation of podocin in a dose-dependent manner decreases the interactions between nephrin, podocin, and CD2-assocaited protein (CD2AP) in cultured podocytes [49]. SEMA3A induced a 10-fold increase in podocyte apoptosis by decreasing the Akt survival pathway [49]. Excess SEMA3A was shown to induce endothelial cell swelling and thickening, lamination of the GBM, and podocyte-foot-process effacement, all of which were transient and reversible upon withdrawal of transgene induction. SEMA3A disrupted podocyte shape in an autocrine fashion, based on podocyte contraction and F-actin collapse [69]. It has been reported that the mechanism under the GBM phenotype change was through increased matrix metalloproteinase 9 expression or the composition of the collagen and laminin chain [47]. Excess SEMA3A caused reversible nephrin downregulation, while the podocin expression and WT1^+^ nuclei counts were not altered, suggesting that SEMA3A caused decreased nephrin expression without podocyte loss. In addition, GST-binding assays revealed a direct interaction between plexin A and nephrin [69], indicating that extracellular SEMA3A signaling may be directly linked to the slit-diaphragm signaling complex. Increased SEMA3A mRNA and protein expression were found in experimental models of puromycin (PAN)-induced podocyte injury [57] (Table 1). In our previous study, we investigated the pathological roles of SEMA3A signaling on podocyte injury using a doxorubicin (Dox)-induced podocytopathy mouse model and examined the therapeutic effect of a SEMA3A inhibitor, SM-345431 [70] (Table 2). We indicated that Dox induced massive albuminuria and podocyte apoptosis via JNK signaling, as well as an increase in SEMA3A expression in podocytes, all of which were improved by treatments with SM-345431 [46]. We also examined serum and urinary SEMA3A levels in 72 patients who underwent kidney biopsies and showed that urinary SEMA3A levels in minimal-change nephrotic syndrome (MCNS) patients were higher compared to other patients [59]. Furthermore, we evaluated their urinary SEMA3A and MCNS activity, and found that levels of urinary SEMA3A at onset were significantly higher than those at remission in patients with MCNS. These results suggested that the urinary SEMA3A might be useful as a biomarker for MCNS.

Diabetic nephropathy (DN) is one of important complications of DM [71], and is a leading cause of CKD and end-stage renal disease (ESRD) worldwide [72,73]. DN is known to cause podocyte injury and generally starts with microalbuminuria, which progresses to GBM thickness, mesangial expansion, macroalbuminuria, and, finally, a decreasing glomerular filtration rate [74,75]. A multifactorial interaction of factors is involved in DN, such as the formation of advanced glycation end products (AGEs), and the renin–angiotensin system (RAAS), further stimulating protein kinase C, and the generation of reactive oxygen species (ROS) [76,77] and microRNAs. It has been reported that urinary SEMA3A excretion was increased early after the induction of diabetes in diabetic mouse models and in diabetic patients with albuminuria, particularly in those with macroalbuminuria [60]. In diabetic mice, podocyte-specific SEMA3A overexpression (SEMA3A^+^) caused Kimmelstiel-Wilson-like nodular glomerulosclerosis, massive proteinuria and kidney insufficiency [42]. Increased SEMA3A expression was found in db/db kidneys [57], as well as streptozotocin-induced-diabetes mouse kidneys [61]. The genetic ablation and inhibition of SEMA3A signaling ameliorated diabetes-induced kidney dysfunction [60]. Importantly, a SEMA3A inhibitor, xanthofulvin treatment or the deletion of podocyte plexin A1 abrogated diabetic nodular glomerulosclerosis induced by the SEMA3A^+^ gain of function [42]. A recent study revealed that microRNAs play important roles in DN pathogenesis. miR-15b-5p restored cell proliferation in high-glucose-induced podocytes by downregulating proapoptotic protein markers, Bax and cleavaged caspase-3, and upregulating the antiapoptotic protein Bcl-2 [78]. miR-15b-5p remarkably decreased the high-glucose-induced inflammatory response via the downregulation of cytokines, IL-1β, TNF-α and IL-6. In addition, it was reported that SEMA3A is a direct target of miR-15b-5p, and the beneficial effects of miR-15b-5p were impeded by excess SEMA3A [78]. SEMA3A is also targeted by miR-23b-3p [79] and miR-16-5p [80]. KCNQ1 opposite-strand/antisense transcript 1(KCNQ1OT1), a long non-coding RNA (lncRNA), was recognized as a miR-23b-3p sponge, and KCNQ1OT1 inhibition ameliorated DN by absorbing miR-23b-3p and regulating SEMA3A [79]. It has also been reported that serum lncRNA T-cell factor7 (TCF7) were elevated in patients with DN and TCF7 silencing ameliorated high-glucose-induced podocyte injury by downregulating SEMA3A via miR-16-5p [80]. Collectively, excess SEMA3A is involved in the progression of DN, and SEMA3A targeting may be a potential therapy against DN.

### 4.2. Acute Kidney Injury

AKI is recognized as a major public health problem, affecting millions of patients worldwide and leading to higher mortality [81], CKD progression, and sometimes to the new onset of CKD, called the AKI–CKD transition. Kidney ischemia-reperfusion injury (IRI), nephrotoxic agents such as LPS or cisplatin, infection leading to sepsis, and contrast-related injury are major causes of AKI [82]. Currently, AKI is diagnosed according to serum creatinine levels and urine volume [83]. Early prediction before an increase in serum creatinine levels and early treatment are important for patients at risk of AKI. Urinary SEMA3A has been shown to be increased within 6 h after IRI, whereas serum creatinine is increased at 24 h in animals [43]. Urinary SEMA3A was also shown to increase, and peaked at 2 h, after liver-transplantation-induced AKI [84]. Serum SEMA3A in cisplatin-induced AKI was upregulated at 24 h and 48 h. In pediatric patients, AKI was detected 48 h after a cardiopulmonary bypass (CPB) through serum creatinine levels [43], while urine SEMA3A was elevated 2 h after CPB and peaked at 6 h. Moreover, an early increase in urinary SEMA3A levels were associated with clinical outcomes, such as the severity of AKI and the length of a hospital stay [43]. Urinary SEMA3A was compared with other urinary biomarkers, IL-18 [85], L-type fatty acid-binding protein(L-FABP) [86], gelatinase-associated lipocalin (NGAL) [87] and N-acetyl-β-d-glycosaminidase (NAG), in intensive care unit (ICU) admission. These biomarkers showed similar performance in detecting established AKI, later-onset AKI and AKI progression, while urinary SEMA3A was not increased in non-progressive established AKI. Finally, urinary SEMA3A was not increased in sepsis-induced AKI, while levels of other urinary biomarkers were increased [88,89]. An increase in urinary SEMA3A was also reported in contrast-induced acute kidney injury [63]. Among 168 patients who underwent percutaneous coronary intervention (PCI), 20 patients developed AKI. Both urinary SEMA3A and NGAL levels were significantly elevated at 2 h and 6 h post-PCI procedure, and peaked at 2 h post-PCI in the AKI patients, which was much earlier than the rise in serum creatinine levels at 48–72 h post-PCI. Further receiver operating characteristic (ROC) analyses of SEMA3A at 2 h after PCI showed a better predictive sensitivity and specificity compared to NGAL. These results indicate that urinary SEMA3A may be useful as an early and predictive biomarker for AKI.

In addition to biomarkers, SEMA3A signaling is also expected to be a therapeutic target. SEMA3A expression was increased after LPS-induced AKI in mouse tubular epithelial cells, as well as in an LPS-treated rat-kidney-proximal-tubular-epithelial-cell line in vitro via Rac1/nuclear factor kappa-light-chain enhancer of activated B cells (NF-κB) p65 and JNK pathways [45]. In addition, the inhibition of SEMA3A by (-)-epigallocatechin-3-gallate (EGCG) could significantly ameliorate LPS-induced kidney inflammation and apoptosis [45]. It is also reported that genetic silencing and the pharmacological inhibition of SEMA3A ameliorated kidney injury from IRI by inhibiting inflammation and epithelial cell apoptosis [62]. These observations indicate an underling signaling pathway of SEMA3A and the potential utility of a SEMA3A inhibitor as a therapeutic agent for regulating inflammation and apoptosis in AKI. G-protein-coupled receptors (GPCRs) are known to participate in plenty of physiologic functions, and some GPCRs have critical roles in the regulation of kidney function. Among them, Gpr97 is a newly identified adhesion GPCR. Gpr97 was upregulated in IRI-induced AKI mice kidneys [90]. Both in vivo and vitro study have revealed that Gpr97 deficiency attenuated AKI-induced kidney injury by regulating SEMA3A signaling. It was also reported that curcumin, well known for its antioxidant and anti-inflammatory properties, and 12/15 lipoxygenase inhibitor-LOXblock-1 ameliorated IRI-induced AKI by reducing inflammatory processes, oxidative stress and apoptosis, and the effects were through the suppression of the SEMA3A signaling pathway [91]. Another study highlighted the protective effects of human-bone-marrow-derived mesenchymal stem cell exosomes in kidney IRI by delivering miR-199a-3p to kidney cells [92]. The mechanism involved downregulating Sema3A expression and activating Akt and extracellular signal-regulated kinase (ERK) signaling pathways, ultimately leading to reduced apoptosis and improved kidney function. These reports indicate a potential avenue for developing new therapeutic strategies to target SEMA3A signaling for AKI.

### 4.3. Chronic Kidney Disease

CKD is characterized by progressive damage and a loss of kidney function, in which parenchymal cell loss, chronic inflammation, fibrosis and the reduced regenerative capacity of the kidney are involved in its progression [93]. It was reported that urinary SEMA3A levels were positively correlated with the urine albumin-to-creatinine ratio and serum creatinine levels in hypertensive patients [94]. In the study, patients with CKD showed higher urinary SEMA3A levels compared to those without CKD. Kidney fibrosis is the common pathological pathway of kidney diseases. In our previous study, we evaluated SEMA3A signaling by usingunilateral ureteral obstruction (UUO) mouse model, a kidney fibrosis model [64]. After UUO surgery, SEMA3A expression in the proximal tubular area and NRP1 expression in the fibroblast and tubular cells were increased. The expression of a myofibroblast marker, tenascin-C, and kidney fibrosis were increased in UUO kidneys, all of which were ameliorated by a SEMA3A inhibitor through the regulation of JNK signaling [64]. One of the important mechanisms in kidney tubulointerstitial fibrosis is the kidney tubular epithelial–mesenchymal transition (EMT) process, where kidney tubular epithelial cells lose their cell-to-cell membrane connection and their structural polarity to become a spindle-shaped mesenchymal-like phenotype [95]. Our study indicated that the injection of a SEMA3A inhibitor, SM-345431, could attenuate UUO-induced EMT in vivo [64]. We also demonstrated that recombinant SEMA3A caused tubular cell EMT, and SM-345431 treatment was able to ameliorate TGF-β1-induced EMT in vitro. We also indicated a positive correlation between urinary SEMA3A and a tubular injury marker, urinary NAG, in patients who underwent kidney biopsy. Collectively, SEMA3A signaling may be involved in the progression of kidney fibrosis under CKD, and the inhibition of SEMA3A signaling might be a therapeutic option for protecting a patient from kidney fibrosis [64].

### 4.4. Systemic Lupus Erythematosus

SEMA3A also regulates immune systems, especially enhancing T-cell and B-cell regulatory properties [3]. Hence, it was reported that SEMA3A is involved in the pathogenesis of autoimmune diseases, including SLE [66], rheumatoid arthritis [96] and Sjogren’s syndrome [97]. SLE is a multi-system autoimmune disease characterized by the aberrant activity of the immune system, and presents with a wide range of clinical manifestations including skin, synovia, brain and kidney [98]. Of note, an analysis of SEMA3A immunostaining in kidneys from patients with lupus nephritis (LN) revealed an increase in SEMA3A expression in patients with LN, while SEMA3A expression was negatively associated with clinical–pathological parameters, including proteinuria and kidney function [65]. In contrast, another study indicated that serum SEMA3A levels in SLE patients were lower than in normal individuals [66]. Aiming to establish a regulatory/protective role for SEMA3A in SLE, serum SEMA3A was assessed in patients with SLE, and this level was compared with SLE disease activity [66], where serum SEMA3A levels were lower in SLE patients compared to those in normal controls. In addition, altered serum SEMA3A levels were found to be inversely correlated with SLE disease activity, mainly with kidney damage and the presence of anti-cardiolipin antibodies. These findings suggest an important role of SEMA3A in SLE.

It was reported that SEMA3A downregulated autoimmune responses by suppressing the over-activity of both B and T cells [99,100]. SEMA3A levels in the B-regulatory cells of patients with SLE were smaller compared to those of normal individuals. Toll-like receptor (TLR)-9 expression could possibly be modulated in the memory B cells of SLE patients, which is associated with the production of inflammatory cytokines such as IL-6 and anti-dsDNA [101,102]. SEMA3A co-cultured with purified B cells from SLE patients significantly reduced TLR-9 expression, supporting the idea that SEMA3A may regulate B-cell autoimmunity in SLE [66]. Serum SEMA3A levels were decreased in SLE while increased in rheumatoid arthritis and Sjogren’s syndrome, compared to healthy controls. How about urinary SEMA3A levels? A study analyzed urinary SEMA3A levels in 38 patients with SLE [103]. Among them, 13 patients had kidney involvement. Urinary SEMA3A levels were lower in SLE patients compared to healthy volunteers, and especially lower in SLE patients with LN than in patients without nephritis, indicating that urinary SEMA3A is inversely correlated with proteinuria and SLE disease activity. The aberrant expression of SEMA3A urine and serum levels in SLE may suggest important roles of SEMA3A in SLE disease activity. Indeed, it was reported that SEMA3A injections in a New Zealand black (NZB)/W mice model of LN delayed the appearance of proteinuria and reduced kidney damage, as well as causing a decrease in immune complex deposition in the glomeruli, indicating the protective effect of SEMA3A in LN [104].

**Table 2:** Kidney outcome in reports targeting SEMA3A.

Etiology	Model	SEMA3A- Targeting	Targeting Method	Species	Outcome	Target	Function	Ref.
Podocyte injury	Dox	Down	SEMA3A inhibitor	Mice	Proteinuria ↓	JNK	Anti-apoptosis	[70]
-	-	Up	Recombinant SEMA3A	Mice	Nephrotic proteinuria	-	Podocytopathy	[44]
-	-	Up	Podocyte SEMA3A^+^	Mice	Proteinuria ↑	Dysregulation of nephrin, MMP9, αvβ3 integrin	Podocytopathy	[69]
DN	STZ	Down	SEMA3A^-^	Mice	Proteinuria ↓	-	-	[60]
	STZ	Down	SEMA3A inhibitor	Mice	Proteinuria ↓ Kidney fibrosis ↓ Kidney dysfunction ↓	-	-	[60]
	High- glucose	Down	miR-15b-5p	Podocyte	-	-	Anti-apoptosis Anti-inflammation	[78]
	High- glucose	Up	KCNQ1OT1	Podocyte	-	miR-23b-3p	Inflammation Apoptosis	[79]
	High- glucose	Down	TCF7 silence/SEMA3A-siRNA	Podocyte	Cytotoxicity↓	miR-16-5p	-	[80]
	Podocyte SEMA3A^+^ & STZ	Down	SEMA3A inhibitor (xanthofulvin)	Mice	Proteinuria ↓ Kidney dysfunction ↓	MICAL1	-	[42]
AKI	IRI	Down	SEMA3A-/SEMA3A-inhibitor	Mice	Kidney dysfunction ↓ Neutrophil infiltration ↓	-	Anti-apoptosis, Anti-inflammation	[62]
	IRI/cisplatin	Down	Gpr97-	Mice	Kidney dysfunction ↓ Neutrophil infiltration ↓	Hour	Anti-apoptosis, anti-inflammation	[90]
	IRI	Down	LOXblock-I/Curcumin	Rats	-	-	Anti-apoptosis, Anti-inflammation	[91]
	IRI	Down	miR-199a-3p	Mice	Kidney dysfunction ↓	Akt/ERK pathway	Anti-apoptosis	[92]
	LPS	Down	EGCG	Mice	Kidney dysfunction ↓ Neutrophil infiltration ↓	Rac1/NF-κB p65/JNK pathway	Anti-apoptosis, Anti-inflammation	[45]
CKD	UUO	Down	SEMA3A Inhibitor (SM-345431)	Mice	Kidney fibrosis ↓	JNK	Anti-apoptosis, Anti-EMT	[64]
LN	NZB	Up	Recombinant SEMA3A	Mice	Proteinuria ↓ Immune complex- Deposition ↓	-	-	[104]

DN: diabetic nephropathy; AKI: acute kidney injury; CKD: chronic kidney disease; LN: lupus nephritis; SEMA3A: semaphorin3A; Dox: doxorubicin; STZ: streptozotocin; IRI: ischemia-reperfusion injury; LPS: lipopolysaccharide; UUO: unilateral ureteral obstruction; NZB: New Zealand black mouse; JNK: c-Jun N-terminal kinase; MMP9: matrix metalloproteinase 9; MICAL1: Molecules interaction with CasL1; HuR: RNA-binding protein human antigen R; Akt: activated the protein kinase B; ERK: extracellular signal regulated kinase pathways; Rac1: Ras-related C3 botulinum toxin substrate 1; NF-κB: nuclear factor kappa-light-chain enhancer of activated B cells; EMT: epithelial-mesenchymal transition; KCNQ1OT1: KCNQ1 opposite strand/antisense transcript 1; TCF7: long non-coding RNA T-cell factor 7; EGCG: (-)-epigallocatechin-3-gallate. Up arrows: increase. Down arrows: decrease.

## 5. SEMA3A and Cardiorenal Syndrome

Cardiorenal syndrome (CRS) is a complex interaction between the heart and kidneys, where dysfunction in one organ causes or exacerbates dysfunction in the other [105]. It was reported that levels of SEMA3A were increased in post-infarcted rat hearts [106]. In the study, intravenous SEMA3A administration improved cardiac autonomic regulation, while the infarct size and cardiac function were not affected. It was also reported that SEMA3A expression in circulating monocytes was increased in patients 30 days after myocardial infarction [107]. In the report, SEMA3A reduced cardiac inflammation after myocardial ischemia in mice. Although further studies are still required to conclude, SEMA3A signaling might be involved in the CRS after myocardial infarction.

## 6. Conclusions

**Figure 2:**
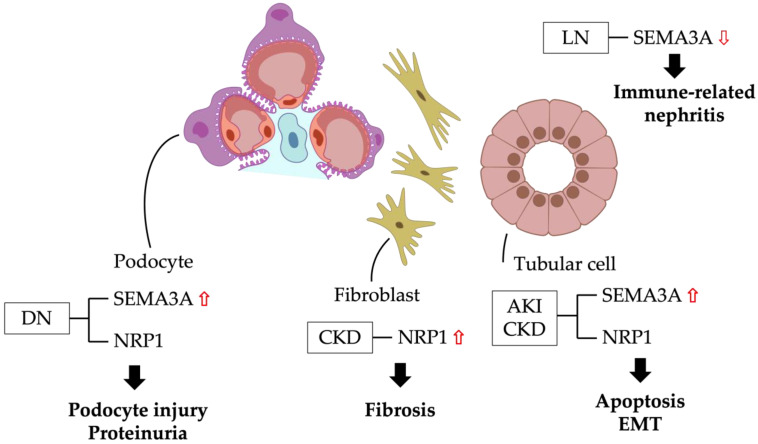
SEMA3A and pathophysiology in kidney diseases. DN: diabetic nephropathy; CKD: chronic kidney disease; AKI: acute kidney injury; LN: lupus nephritis; EMT: epithelial–mesenchymal transition; SEMA3A: semaphorin3A; NRP1: neuropillin-1. Red up arrows: increase. Red down arrow: decrease.

We have summarized the current knowledge regarding the role of SEMA3A in kidney pathophysiology and its potential use in kidney diseases. On the basis of these studies, SEMA3A plays an important role in kidney morphogenesis and kidney diseases. SEMA3A loss-of-function studies indicate that SEMA3A is required for the maintenance of structure and function in the glomerular filtration barrier. In contrast, excess SEMA3A causes the progression of a variety of kidney diseases, including DN, AKI and CKD, through an increase in albuminuria, kidney fibrosis, apoptosis and inflammation. Current speculations around SEMA3A and the pathophysiology of kidney diseases is summarized in Figure 2. Increased SEMA3A expression from tubular cells may cause tubular apoptosis and EMT in an autocrine manner under AKI and CKD, as well as cause fibroblast activation. In addition, increased SEMA3A expression from podocytes may cause podocytopathy, leading to proteinuria in DN. Therefore, SEMA3A-targeting therapy may be a novel therapeutic option for treatment against a variety of kidney diseases, including AKI, CKD and proteinuric diseases. Indeed, SEMA3A-mutant mice or the pharmacological-based inhibition of SEMA3A protected from these kidney diseases, suggesting the potential of SEMA3A inhibitory treatment in clinic use in the future. On the other hand, SEMA3A deficiency may lead to the progression of LN through upregulating autoimmune responses by the activation of T cells and B cells, suggesting a concern of side effects of the inflammatory response in kidneys under SEMA3A inhibitory therapy. In addition, the detailed mechanisms of SEMA3A underlying kidney diseases are not fully understood;, there are several possible signaling pathways, involving SEMA3A signaling, such as Rac1/NF-κB p65, JNK and TLR4 signaling, which may regulate inflammation and cell apoptosis. Of note, the Rho family of GTPases acts downstream of plexin A, regulating adhesion, proliferation, migration and survival in different cell types, and interacts with diverse signaling pathways, suggesting that the Rho family may be involved as a downstream effector of SEMA3A. We previously proposed a possible connection between the SEMA3A–NRP1–plexin A1 complex and JNK signaling with FERM, ARH/RhoGEF and the pleckstrin domain protein 2 (FARP2)/Rac1/Mixed-LineageKinase3 (MLK3)/mitogen-activated protein kinase (MKK) 4/7 cascade [64]. Further studies are still needed for a deep understanding of SEMA3A signaling.

## Data Availability

There were no datasets generated during and/or analyzed during the current study.
